# Cell type–specific extracellular matrix guided the differentiation of human mesenchymal stem cells in 3D polymeric scaffolds

**DOI:** 10.1007/s10856-017-5912-9

**Published:** 2017-05-22

**Authors:** Yong Mao, Tyler Hoffman, Amy Wu, Ritu Goyal, Joachim Kohn

**Affiliations:** New Jersey Center for Biomaterials, Rutgers University, 145 Bevier Rd., Piscataway, NJ 08854 USA

## Abstract

**Abstract:**

The tissue microenvironment has profound effects on tissue-specific regeneration. The 3-dimensional extracellular matrix (ECM) niche influences the linage-specific differentiation of stem cells in tissue. To understand how ECM guides tissue-specific regeneration, we established a series of 3D composite scaffolds containing ECMs derived from different primary cells isolated from a single animal species and assessed their impact on the differentiation of human mesenchymal stem cells (hMSCs). Synthetic microfiber scaffolds (fiber mats) were fabricated by electrospinning tyrosine-derived polycarbonates (pDTEC). The bovine primary fibroblasts, chondrocytes and osteoblasts cultured on the fiber mats produced and assembled their ECMs, infiltrating the pores of the fibrous scaffold. The composite scaffolds were decellularized to remove cellular components, preserve ECM and minimally affect polymer integrity. Characterization of the ECMs derived from different primary cells in the composite scaffolds showed overlapping but distinct compositions. The chondrogenic and osteogenic differentiation of hMSCs on the different composite scaffolds were compared. Our results showed that ECM derived from chondrocytes cultured in synthetic fiber mats promoted the chondrogenic differentiation of hMSC in the presence or absence of soluble inducing factors. ECM derived from co-culture of osteoblasts and chondrocytes promoted osteogenic differentiation in hMSCs better than ECM derived from chondrocytes. This study demonstrated that decellularized ECMs derived from different cell types formed within synthetic fiber scaffolds guide the tissue-specific differentiation of hMSCs. These composite scaffolds may be developed into models to study the mechanisms of ECM-induced tissue regeneration.

**Graphical Abstract:**

## Introduction

Scaffolds are often required to support tissue repair, regeneration, or reconstruction following the loss of tissue caused by injury or disease [1]. Scaffolds can be categorized as biological, synthetic or a combination of both termed here as composite. Biological scaffolds are either generated through decellularization of tissue/organs or fabricated from purified extracellular matrix (ECM) components [2, 3]. The advantage of biological scaffolds is the preservation of biological activity, which regulates cell functions and guides tissue regeneration [4]. The disadvantage of decellularized tissue scaffolds is the limitation on donor tissue availability, sample uniformity, and compromised mechanical properties following decellularization [5]. Synthetic scaffolds can be designed and tailored to match the structure and mechanical properties of native tissue matrices, which makes them very promising candidates for tissue engineering [6, 7]. However, the lack of the biological activity in synthetic scaffolds remains a challenge for inducing tissue-specific regeneration. The recent development of composite scaffolds, which incorporates the ECM components or decellularized ECM matrix into synthetic scaffolds, often shows improved biological activity [8–10].

Tissue-specific regeneration is the ultimate goal of tissue repair. Numerous studies have demonstrated that tissue microenvironments possess unique mechanical and biological signals, regulate cellular behaviors and influence tissue regeneration [11–13]. Marinkovic et al. analyzed the ECMs derived from bone marrow (BM) stem cells or adipose (AD) stem cells cultured on 2D surfaces [11]. The BM- and AD-specific ECMs preferentially directed MSC differentiation towards osteogenic or adipogenic lineage, respectively [11]. These experiments provided the evidence that ECMs derived from cells of different origins can influence the fate of stem cells. Since BM or AD cells were not terminally differentiated cells, which more accurately mimics tissue matrices, differentiated cell populations responsible for producing ECM within mature tissues should be considered. In addition, ECM deposition is regulated differently on 2D verses 3D culture conditions [14, 15]. ECM assembled by cells on a 2D surface may not resemble the ECM assembled in a 3D environment in situ. Therefore, the ECM assembled by differentiated primary cells on a 3D culture substrate, such as a fiber mat, is more likely to mimic the ECM in the tissue microenvironment.

Poly(desamino tyrosyl tyrosine ethyl ester carbonate) (pDTEC) belongs to a family of biocompatible polymers with tunable degradability [16, 17]. Electrospinning poly(desamino tyrosyl tyrosine ethyl ester carbonate) (pDTEC) into fibrillar scaffolds was shown to support cell growth [18, 19]. In this study, pDTEC fiber mats were fabricated and used as the synthetic base to support the in vitro synthesis of ECM by different types of cells.

Chondrocytes, fibroblasts and osteoblasts are the primary cells that produce the ECM for cartilage, dermis and bone tissues, respectively. In order to recapitulate an ECM environment that is a simplified model of a tissue environment, primary cells freshly isolated from the tissue of interest were cultured within the synthetic fibrillar scaffolds. Unlike the previous reports, in which either primary cells at a later passage or cells isolated from different species were used [8, 20], our study used the primary cells isolated from a range of mature tissues from a single animal species to produce ECM in a 3D culture system. Cells (P0-P1) were seeded on fiber mats and cultured until confluent. The composite fiber mats were then prepared by decellularization, removing the primary cell population, retaining only the cell-derived ECM.

The decellularized ECMs derived from cells with different tissue origins in fiber mats were characterized and showed overlapping but distinct compositions. Differentiation of hMSCs grown on the different composite fiber mats were compared. Our data showed that the ECM derived from chondrocytes promoted chondrogenic differentiation of hMSCs and the ECM from osteoblasts promoted osteogenic differentiation of hMSCs. These observations indicated that ECM derived from different primary cell populations grown within a synthetic fibrillar scaffold may possess tissue-specific signals and guide tissue-specific differentiation. Our composite fiber mats may provide a model system to understand the roles of ECMs or individual ECM components in tissue-specific differentiation and may eventually be developed into bioactive scaffolds to support tissue-specific regeneration.

## Materials and methods

### Isolation of primary cells from bovine tissues

Full-thickness skin, knee joints and long bones of 1–2 month old calves were obtained from Farmtopharm, LLC. (Warren, NJ). Tissues were aseptically processed and the isolation of fibroblasts, articular chondrocytes or osteoblasts was performed following the standard protocols [21].

### Preparation of electrospun fibrillar scaffolds (fiber mats)

The 3D fibrous scaffolds (fiber mats) made of poly(desamino tyrosyl tyrosine ethyl ester carbonate) (pDTEC) were fabricated by electrospinning from tetrahydrofuran (THF) and N,N’-dimethylformamide (DMF) mixed solution (9:1) as described previously [17, 18, 22].

### Preparation of composite ECM-fiber mats by decellularization

The fiber mats were cut into 10 mm diameter discs and sterilized by UV treatment on each side for 2 h and placed into the wells of 48-well plates. Bovine fibroblasts, articular chondrocytes or osteoblasts at passage 1 were seeded onto each fiber mat at 1 × 10^4^/well in 48-well tissue culture plates. When the cells reached confluence, the fiber mats were decellularized using a modified protocol [14]. Briefly, the fiber mats were washed twice with 1 ml PBS and freeze/thaw cycled at −80°C/37°C three times. The fiber mats were then washed with serial buffers as described previously [14]. The decellularized fiber mats were stored in PBS at 4°C until used.

### Scanning electron microscopy (SEM)

Fiber mats were dried in a vacuum oven overnight at room temperature. The fiber mats were sputter-coated with gold/palladium (SCD004; 30 mA; 120 s). The coated samples were analyzed using a scanning electro microscope (Amray1830I, 20 kV).

### Quantification of DNA

The DNA in the fiber mats with or without decellularization was extracted using proteinase K digestion followed by phenol:chloroform:isoamylalcohol extraction [23]. The recovered DNA was quantified using Quant-iT^™^ PicoGreen^®^ dsDNA (Invitrogen) assay as per the manufacturer’s protocol.

### Quantification of sulfated glycosaminoglycans (sGAG)

To extract sGAG, 0.2–0.5 ml of papain extraction buffer (0.1 mg/ml papain (Sigma-Aldrich, from papaya latex), 0.2 M sodium phosphate buffer, 0.5 M EDTA (pH 7.0), 400 mg cysteine HCl) was added to wells containing cells. The lysates were transferred to Eppendorf tubes and incubated at 65˚C for overnight. After extraction, sGAG levels were measured using Blyscan Assay Kit (Biocolor, UK) following manufacturer’s instructions.

### Staining of cells with Alcian Blue

Primary cells (1 × 10^4^/well) were seeded to the wells of tissue culture treated 48-well plate and cultured in DMEM medium containing 10% FBS and 50 µg/ml ascorbic acid for 3 days. The cells were fixed with 4% paraformaldehyde in PBS (Thermo Fisher Scientific) for 15 min. Fixed cells were washed with PBS and stained with 1% Alcian blue (Sigma) in 3% acetic acid for 30 min. After staining, cells were thoroughly washed with PBS.

### Characterization of protein compositions in ECM-fiber mats

The decellularized ECM-fiber mats were lysed in 2xSDS sample buffer (120 mM Tris-HCl pH 6.8, 10% Glycerol, 3% SDS, 0.2 M DTT, 0.004% bromophenol blue). Samples were boiled and loaded onto two 4–20% gradient SDS-gel (Bio-Rad) in parallel. After SDS-PAGE, one gel was stained using silver staining kit (Silver Quest, Invitrogen) following the manufacture’s protocol. The other gel was transferred to a nitrocellulose membrane and Western blotting was performed using antibodies against fibronectin at 1:1000 dilution (R457, Princeton University) or bovine type II collagen at 1:500 dilution (Abcam, ab3092) [24].

### Differentiation of human mesenchymal stem cells

The bone marrow derived human mesenchymal stem cells (hMSCs, donors #7076L and #8001L) were obtained from Texas A&M Institute for Regenerative Medicine (Temple, TX). hMSCs (Passage 2–4) were cultured in hMSC growth medium: MEM-alpha medium (Gibco) supplemented with 10% fetal bovine serum (Atlanta Biologicals, Inc. Flowery Branch, GA). When the cells reached to 80–90% confluence, hMSCs were detached using 0.05 % trypsin plus EDTA (Lonza), re-suspended in hMSC growth medium, and used for the following chondrogenic or osteogenic induction experiments. hMSC suspensions with the passage number 3–5 (5–10 × 10^4^ cells/mat) were directly seeded onto each fiber mat, which was held in place by an O-ring (McMaster-Carr) in a well of a 48-well plate. After 24 h, the O-rings were removed and fiber mats were transferred to fresh wells of a 48-well plate. For chondrogenic induction, serum-free hMSC chondrogenic SingleQuots™ medium (Lonza) containing insulin/transferring/selenium (ITS), dexamethasone, ascorbate, sodium pyruvate, proline, GA-1000, L-glutamine was used. The transforming growth factor TGF-β3 (Lonza) was freshly added at 10 ng/ml to the cells for 14 days. For osteogenic induction, serum-free hMSC Osteogenic SingleQuots™ medium (Lonza) was used for 14 days. The un-induced groups were maintained with hMSC growth medium for 14 days. The medium was changed every 3 days for all culture conditions.

### Quantitative PCR (qPCR) analysis

hMSCs on fiber mats were lysed by 0.2 ml/well of RNA lysis buffer (Promega). The lysates were centrifuged in a microcentrifuge at 6500 *g* for 5 min. The supernatants were used for RNA extraction/purification using SV 96 Total RNA Isolation System following manufacturer’s protocol (Promega). RNA concentration and purity was measured using Nanodrop2000 (Thermo Scientific). 200 ng of RNA from each sample was used for cDNA synthesis.

The primers used for qPCR were the following: Quanti-tech GAPDH (QT01192646), SOX 9 (QT00001498), COL1A1 (QT00037793) and COL2A1 (QT00998844) were purchased from Qiagen (Valencia, CA). The qPCR analysis was performed using Roche LightCycler 480 as described [25]. Every sample was run in triplicate. After the run was completed, a second derivative analysis was performed using the raw data to determine the mean Cp for each sample. GAPDH expression served as an internal control. The relative mRNA expression was determined by Pfaffl analysis (E^ΔCp^ target/E^ΔCp^ reference) in which primer efficiency E = 10^^^(−1/slope) and ΔCp = mean Cp of sample - mean Cp of the experimental control. The relative gene expression (fold) was normalized to the expression of each gene in the undifferentiated hMSCs (set as 1). The ratio of COL2A1/COL1A1 was expressed as 2^^^−(Cp^COL2A^−Cp^GAPDH^)/2^^^(Cp^COL1A^−Cp^GAPDH^) [26].

### Statistical analysis

Results of each independent experiment were based on repetitive samples (*n* ≥ 3) and data were expressed as the mean ± standard deviation. One-way ANOVA with a Tukey’s multiple comparisons test was used to determine the statistical significance. Differences were considered significant at a p value of <0.05.

## Results

### Isolation and characterization of primary cells

**Fig. 1:**
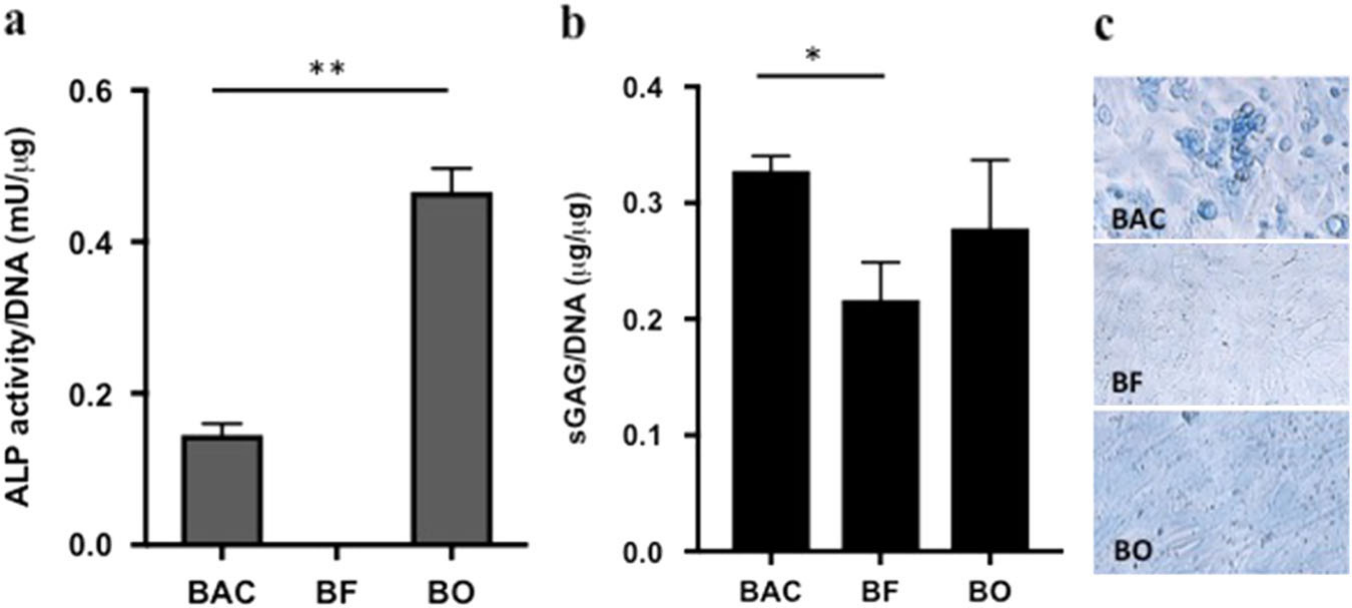
Characterization of isolated primary bovine cells cultured on tissue culture plates for 3 days. **a** ALP activity normalized to DNA. **b** Quantification of sGAG normalized to DNA. Data are presented as mean ± SD (*n* = 3, *p < 0.05 and **p < 0.01). **c** Alcian blue staining of P1 cells on tissue culture plates

In order to mimic tissue ECMs, cells producing ECM within mature tissues are chosen for this study. Primary skin fibroblasts (BF), articular chondrocytes (BAC) and osteoblasts (BO) were isolated from bovine tissues following the standard isolation procedure developed for each cell type as described in Materials and Methods. To characterize the isolated cells, cells at Passage 1 were seeded onto tissue culture treated polystyrene (TCP) for 3 days. The alkaline phosphatase (ALP) activities of these cells were measured using ALP activity assay kit (Fig. 1a). There was no ALP activity detected in fibroblasts. The ALP activity was significantly higher in BO cells than in BAC cells (*n* = 3) *p* < 0.01, which is consistent with the characteristics of these cell types. The sulfated glycosaminoglycans (sGAG) in the cells were quantified. As shown in Fig. 1b, BAC produced significantly more sGAG than BF (*n* = 3) *p* < 0.05. The cells were also fixed and stained Alcian blue. Consistent with the quantification analysis, the sGAG contents were more prominent in BAC and BO cells than BF cells (Fig. 1c). If these cells were not pure population of fibroblasts, chondrocytes or osteoblasts, they can be considered as fibroblast-, chondrocyte- or osteoblast-enriched cell populations.

### Preparation and characterization of ECM/fiber mats

To create 3D polymer scaffolds composited with different ECMs, BAC or BF cells at passage 1 were seeded onto fiber mats. The growth of cells on the mats was monitored by alamarBlue assay every other day. When the increase of metabolic activity of cells in fiber mat stalled, the cells in mats were considered to be confluent and the fiber mats were ready to be decellularized. Quantification of DNA in the fiber mats containing BAC or BF before decellularization indicated that, on average, there were more cells in BAC-fiber mat (3.29 ± 0.92 µg/mat) than BF-fiber mat (2.85 ± 0.64 µg/mat) at confluence. However, there was no significant difference between these two cell-fiber mats.

**Fig. 2:**
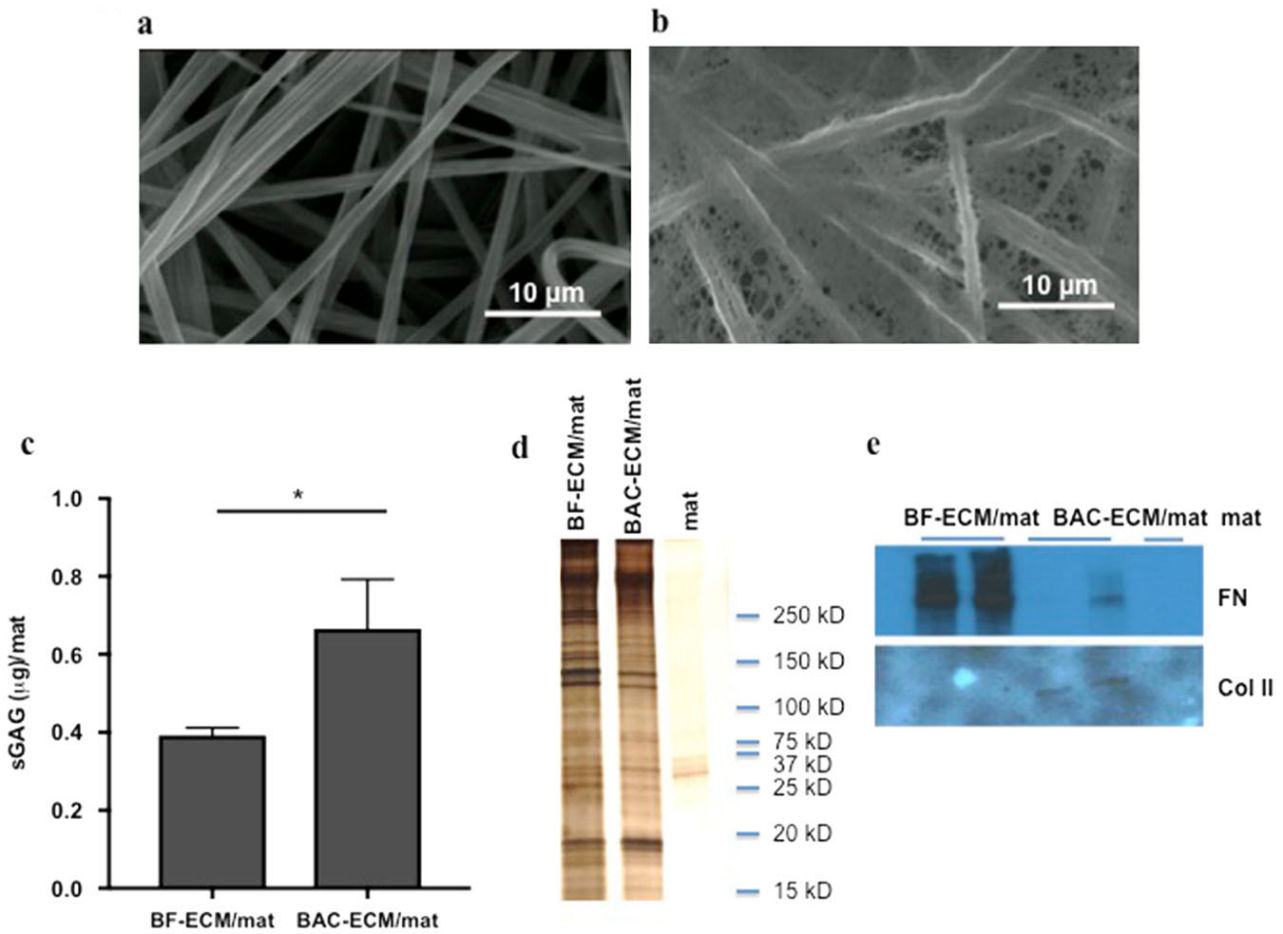
Characterization of ECM/polymer composite fiber mats. Electrospun fiber mats made of pDTEC was analyzed using SEM **a**. BACs were cultured on fiber mats to confluence. The mat was decellularized and analyzed using SEM **b**. **c** The sGAG contents in BF-ECM/mat and BAC-ECM/mat were measured using Blyscan Assay Kit. Data are presented as mean ± SD (*n* = 4, *p < 0.05). **d** The ECM/mats were lysed and analyzed by SDS-PAGE followed by silver staining. **e** The presence of fibronectin (FN) or type II collagen (Col II) in the lysates was analyzed by Western blotting with specific antibodies

The decellularized BAC-fiber mats and BF-fiber mats are referred to as BAC-ECM/mat and BF-ECM/mat, respectively. When the BAC-ECM/mat was analyzed by SEM, an elaborate ECM network was observed in the composite scaffold (Fig. 2b), as compared to the empty mat control (Fig. 2a). The ECM covers the polymer fibers and forms dense networks within the pores between the fibers. The residual sGAG contents in BAC-ECM/mat and BF-ECM/mat were compared. As expected, the sGAG content in BAC-ECM/mat is significantly higher than that of BF-ECM/mat (*n* = 4) *p* < 0.05 (Fig. 2c). The protein compositions of these two composite ECM/mats were analyzed by SDS-PAGE followed by silver staining and Western blotting (Figs. 2d and e). While these two composite mats shared many common protein components, they also had distinct protein compositions. Fibronectin was more enriched in BF-ECM/mat than in BAC-ECM/mat while type II collagen was only detected in BAC-ECM/mat (Fig. 2e). Immunofluorescent staining was performed and further supported the enrichment of fibronectin in BF-ECM mat or the presence of type II collagen in BAC-ECM mat (data not shown). Our results demonstrated that ECM composite scaffolds are readily prepared from different primary cells and they have differential ECM compositions.

### Chondrogenic differentiation of hMSC on ECM/mats

**Fig. 3:**
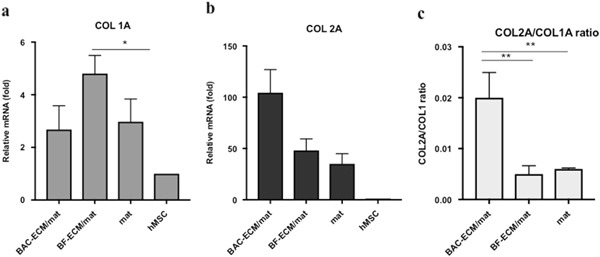
Relative gene expression in hMSCs cultured on scaffolds in the presence of chondrogenic induction. Gene expression is normalized to the starting hMSCs (set as 1) and the housekeeping gene GAPDH. The ratio of COL2A1/COL1A1 (**c**) was calculated from the relative expressions of COL1A1 (**a**) and COL2A1 (**b**) as described in Materials and Methods. Data shown are a representative of two independent experiments. Data are presented as mean ± SD (*n* = 3, *p < 0.05 and **p < 0.01)

**Fig. 4:**
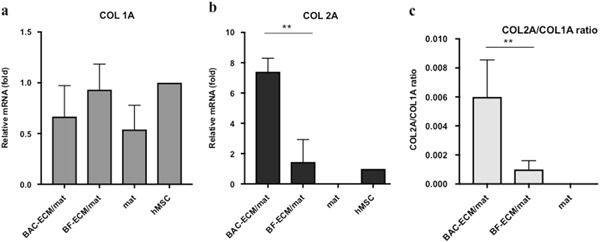
Relative gene expression in hMSCs cultured on scaffolds in the absence of chondrogenic induction. Gene expression is normalized to the starting hMSCs (set as 1) and the housekeeping gene GAPDH. The ratio of COL2A1/COL1A1 (**c**) was calculated from the relative expressions of COL1A1 (**a**) and COL2A1 (**b**) as described in Materials and Methods. Data shown are a representative of two independent experiments. Data are presented as mean ± SD (*n* = 3, **p < 0.01)

In order to study the effect of ECM produced by different cell types on the biological activity of composite scaffolds, the differentiation of hMSC on these fiber mats were compared. In the Presence of chondrogenic soluble factors (TGF-β3), the hMSCs underwent chondrogenic differentiation (Fig. 3). The expression of chondrogenic markers was analyzed by qPCR normalized to the starting hMSC. The expression of type II collagen gene (COL2A1) that of cells in BAC-ECM/mat was significant higher than that of cells in BF-ECM/mat or ECM-free control mat (Fig. 3b; *n* = 3, *p* < 0.01). The expression of SOX9 gene in hMSC on different mats showed a similar trend to the expression of COL2A1 (data not shown). On the other hand, the expression of COL1A1 was significantly increased in the cells cultured in BF-ECM/mat compared with ECM-free mats (Fig. 3a). The chondrogenic potential of biomaterials is often expressed as the ratio of COL2A1:COL1A1 expression in hMSC cultured on such biomaterials [9, 26]. In the presence of chondrogenic inducible factors, BAC-ECM/mat showed the highest chondrogenic potential (Fig. 3c). The differentiation of hMSCs was also studied in the absence of soluble chondrogenic factors. In the absence of chondrogenic induction, the expression of COL2A1 in cells cultured in BAC-ECM/mat was stimulated. On the other hand, hMSC expressed low or non-detectable levels of COL2A1 when cultured on BF-ECM/mat or ECM-free mat in the absence of induction (Fig. 4b). The chondrogenic potential of BAC-ECM/mat remained to be the highest one among all mats tested (Fig. 4c. Our results indicated that the fiber mat composited with chondrocyte ECM possesses chondrogenic signals, which not only enhanced the TGF-β3 induced chondrogenic differentiation of hMSC but also stimulated the expression of chondrogenic marker (COL2A1) of hMSC in the absence of inducible factors.

### Osteogenic differentiation of hMSC on ECM/mats

**Fig. 5:**
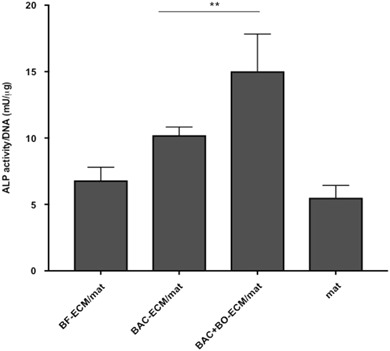
The osteogenic differentiation of hMSCs on ECM/mats. The hMSCs were cultured on control or ECM/mats with osteogenic induction for 14 days. The ALP activities of cells were analyzed and normalized to the DNA of each sample. Data are presented as mean ± SD (*n* = 6 for BAC-ECM/mat and *n* = 3 for all the other groups). **P < 0.01

In order to study the effect of ECM derived from osteoblasts on the differentiation of hMSC, bovine osteoblasts (BO) were cultured in fiber mats. However, BO-ECM composite scaffolds could not be prepared and decellularized using our current method, as the osteoblast-ECM integrated poorly within the pDTEC microfiber mats and tended to lift off the well. Therefore, instead of making osteoblast ECM/mat, BAC and BO cells were co-cultured on fiber mats (seeding ratio 1:1) for 10 days. The decellularized BAC + BO-ECM fiber mats (referred to as BAC + BO-ECM/mat) were prepared. The osteogenic differentiation of hMSC was analyzed by the quantification of ALP activity of cells cultured in different scaffolds with and without decellularized ECM (Fig. 5). The ALP activity was significantly higher in the cells cultured in BAC-ECM/mat than that of cells cultured in ECM-free control mats or BF-ECM/mat (*n* = 6) *p* < 0.05. By incorporating ECM components from osteoblasts, BAC + BO-ECM/mat enhanced the ALP activity of hMSCs even more than that of cells cultured in BAC-ECM/mat. This suggested that ECM composition from osteoblasts contributed osteogenic activity to the ECM/mat.

## Discussion

The composition of native ECM varies between different tissues and organs [27]. To support tissue-specific regeneration, composite scaffolds should contain tissue-specific cues.

In this study, we used isolated primary cells from different bovine tissues to produce composite scaffolds containing tissue-specific ECM on a synthetic fiber mat and demonstrated that these ECMs indeed have differential compositions. Furthermore, these ECMs influenced the differentiation of hMSCs in the presence and absence of growth factors and promoted differentiation towards the linage that the primary cells were isolated from. Our current results indicated that tissue-specific ECM and microenvironment are important to promote tissue-specific stem cell differentiation.

The decellularized ECM-fiber mats developed in this study can simplify the mechanistic studies to address individual ECM components on the tissue-specific guidance. The quantity of particular ECM components in cell-assembled ECMs can be modulated by either the addition of additional exogenous ECM components such as FN or stimulated over expression of proteins such as collagens [28, 29]. By modulating the ECM composition, the role of individual ECM components in influencing the cellular behaviors may be determined.

The composite scaffold fabrication protocol can be easily modified to make composite scaffolds with tunable degradability. Electrospun fiber mats can be prepared using tyrosine-derived polymers with different degradation rates [22]. Composite scaffolds with tuned degradation can be developed to meet specific regeneration requirements of the targeted tissue.

In this study, ECMs derived from primary animal cells (i.e. bovine) drove human MSC to differentiate towards to the linage of the source tissues. This suggests that a possibility to use readily available xenogenic primary cells to make decellularized composite scaffolds for human tissue repair.

We established a simple system to make cell-instructive 3D ECM/polymer composite scaffolds. These composite scaffolds are useful tools to understand tissue-specific regeneration and may guide the design of regenerative scaffolds for clinical uses.

## References

[1] Ko IK, Lee SJ, Atala A, Yoo JJ (2013). In situ tissue regeneration through host stem cell recruitment. Exp Mol Med.

[2] Badylak SF, Brown BN, Gilbert TW, Daly KA, Huber A, Turner NJ (2011). Biologic scaffolds for constructive tissue remodeling. Biomaterials..

[3] Cen L, Liu W, Cui L, Zhang W, Cao Y (2008). Collagen tissue engineering: development of novel biomaterials and applications. Pediatr Res..

[4] Hoshiba T, Lu H, Kawazoe N, Chen G (2010). Decellularized matrices for tissue engineering. Expert Opin Biol Ther.

[5] Liao J, Joyce EM, Sacks MS (2008). Effects of decellularization on the mechanical and structural properties of the porcine aortic valve leaflet. Biomaterials..

[6] Cosson S, Otte EA, Hezaveh H, Cooper-White JJ (2015). Concise review: tailoring bioengineered scaffolds for stem cell applications in tissue engineering and regenerative medicine. Stem Cells Transl Med.

[7] Nair LS, Laurencin CT (2006). Polymers as biomaterials for tissue engineering and controlled drug delivery. Adv Biochem Eng Biotechnol.

[8] Levorson EJ, Hu O, Mountziaris PM, Kasper FK, Mikos AG (2014). Cell-derived polymer/extracellular matrix composite scaffolds for cartilage regeneration, Part 2: construct devitalization and determination of chondroinductive capacity. Tissue Eng Part C Methods.

[9] Levorson EJ, Mountziaris PM, Hu O, Kasper FK, Mikos AG (2014). Cell-derived polymer/extracellular matrix composite scaffolds for cartilage regeneration, Part 1: investigation of cocultures and seeding densities for improved extracellular matrix deposition. Tissue Eng Part C Methods.

[10] Norouzi M, Boroujeni SM, Omidvarkordshouli N, Soleimani M (2015). Advances in skin regeneration: application of electrospun scaffolds. Adv Healthc Mater.

[11] Marinkovic M, Block TJ, Rakian R, Li Q, Wang E, Reilly MA (2016). One size does not fit all: developing a cell-specific niche for in vitro study of cell behavior. Matrix Biol..

[12] Voss A, McCarthy MB, Hoberman A, Cote MP, Imhoff AB, Mazzocca AD (2016). Extracellular matrix of current biological scaffolds promotes the differentiation potential of mesenchymal stem cells. Arthroscopy.

[13] Hoshiba T, Chen G, Endo C, Maruyama H, Wakui M, Nemoto E (2016). Decellularized extracellular matrix as an in vitro model to study the comprehensive roles of the ecm in stem cell differentiation. Stem Cells Int.

[14] Mao Y, Schwarzbauer JE (2005). Stimulatory effects of a three-dimensional microenvironment on cell-mediated fibronectin fibrillogenesis. J Cell Sci.

[15] Liu H, Lin J, Roy K (2006). Effect of 3D scaffold and dynamic culture condition on the global gene expression profile of mouse embryonic stem cells. Biomaterials..

[16] Asikainen AJ, Pelto M, Noponen J, Kellomaki M, Pihlajamaki H, Lindqvist C (2008). In vivo degradation of poly(DTE carbonate) membranes. Analysis of the tissue reactions and mechanical properties. J Mater Sci Mater Med.

[17] Meechaisue C, Dubin R, Supaphol P, Hoven VP, Kohn J (2006). Electrospun mat of tyrosine-derived polycarbonate fibers for potential use as tissue scaffolding material. J Biomater Sci Polym Ed.

[18] Carlson AL, Florek CA, Kim JJ, Neubauer T, Moore JC, Cohen RI (2012). Microfibrous substrate geometry as a critical trigger for organization, self-renewal, and differentiation of human embryonic stem cells within synthetic 3-dimensional microenvironments. FASEB J..

[19] Hsia HC, Nair MR, Mintz RC, Corbett SA (2011). The fiber diameter of synthetic bioresorbable extracellular matrix influences human fibroblast morphology and fibronectin matrix assembly. Plast Reconstr Surg.

[20] Lu H, Hoshiba T, Kawazoe N, Koda I, Song M, Chen G (2011). Cultured cell-derived extracellular matrix scaffolds for tissue engineering. Biomaterials..

[21] Freshney RI. editor Culture of animal cells-a manual of basic techniques. 5th edn. Hoboken, New Jersey. In Wiley-Liss; 2005.

[22] Goyal R, Guvendiren M, Freeman O, Mao Y, Kohn J. Optimization of polymer-ecm composite scaffolds for tissue engineering: effect of cells and culture conditions on polymeric nanofiber mats. J Funct Biomater. 2017;8(1):1–14 10.3390/jfb8010001 PMC5371874 28085047

[23] Fan HaG ML. DNA Extraction from Fresh or Frozen Tissue2000.

[24] Pastino AK, Greco TM, Mathias RA, Cristea IM, Schwarzbauer JE (2017). Stimulatory effects of advanced glycation endproducts (AGEs) on fibronectin matrix assembly. Matrix Biol..

[25] Chen YC, Chen RN, Jhan HJ, Liu DZ, Ho HO, Mao Y (2015). Development and characterization of acellular extracellular matrix scaffolds from porcine menisci for use in cartilage tissue engineering. Tissue Eng Part C Methods.

[26] Steele JA, McCullen SD, Callanan A, Autefage H, Accardi MA, Dini D (2014). Combinatorial scaffold morphologies for zonal articular cartilage engineering. Acta Biomater..

[27] Manabe R, Tsutsui K, Yamada T, Kimura M, Nakano I, Shimono C (2008). Transcriptome-based systematic identification of extracellular matrix proteins. Proc Natl Acad Sci USA.

[28] Green H, Todaro GJ, Goldberg B (1966). Collagen synthesis in fibroblasts transformed by oncogenic viruses. Nature..

[29] Mao Y, Schwarzbauer JE (2006). Accessibility to the fibronectin synergy site in a 3D matrix regulates engagement of alpha5beta1 versus alphavbeta3 integrin receptors. Cell Commun Adhes.

